# Biochemical and molecular identification of Gram-positive isolates with β-hemolysis activity isolated from the nasal swab of pigs during the human meningitis outbreak in Badung Regency, Bali-Indonesia

**DOI:** 10.14202/vetworld.2022.140-146

**Published:** 2022-01-25

**Authors:** K. J. Putra Pinatih, I. W. Suardana, I. D. M. Sukrama, I. B. N. Swacita, R. K. Putri

**Affiliations:** 1 Department of Clinical Microbiology, Faculty of Medicine, Udayana University, Jl. PB. Sudirman Denpasar-Bali, 80234, Indonesia; 2 Department of Preventive Veterinary Medicine, Laboratory of Veterinary Public Health, Faculty of Veterinary Medicine, Udayana University, Jl. PB. Sudirman Denpasar-Bali, 80234, Indonesia; 3 Department of Veterinary Medicine, Faculty of Veterinary Medicine, Udayana University, Jl. PB. Sudirman Denpasar-Bali, 80234, Indonesia.

**Keywords:** 16S ribosomal RNA gene, Gram-positive bacteria, Kit API 20 Strep, nasal of pig, phylogenetic tree

## Abstract

**Background and Aim:**

The nasal cavity of a pig serves as an entry point and a habitat for the colonization of commensal microbes and pathogenic bacteria. Based on biochemical and serological tests, *Streptococcus* b-hemolytic Group C was identified as the Gram-positive bacteria, which resulted in the 1994 outbreak and death of thousands of pigs in Bali. Furthermore, this agent is zoonotic and frequently results in the development of meningitis lesions in the infected pig. Recently, a meningitis outbreak in humans was also reported after the consumption of pig-derived foods at Sibang Kaja, Badung-Bali. This study aimed to identify and characterize Gram-positive β-hemolytic organisms collected from nasal swab of pigs from the outbreak area, as well as to compare API Kit and *16S rRNA* gene analysis methods.

**Materials and Methods:**

This study commenced with the cultivation of two isolates, Punggul Swab Nasal (PSN) 2 and PSN 19, which were characterized by β-hemolysis activity. These samples were then conventionally and molecularly identified using Kit API 20 Strep and 16S ribosomal RNA (rRNA) gene primers, respectively.

**Results:**

Using the Kit API 20 Strep, both isolates were identified as *Enterococcus faecium*, which was previously classified as Group D Streptococci. Based on the *16S rRNA* gene sequencing, PSN 2 and PSN 19 were molecularly confirmed to have 99 and 98.1% similarities with *E. faecium* (NR042054), respectively. Furthermore, both isolates share the same clade in the phylogenetic tree analysis.

**Conclusion:**

Using Kit API 20 Strep and *16S rRNA* gene analysis, the PSN 2 and PSN 9 Gram-positive isolates with β-hemolysis activity from pig nasal swabs were identified as *E. faecium*.

## Introduction

The nasal cavity of a pig is an entry point and a habitat for the colonization of commensal microbes and pathogenic bacteria. Factors such as poor feed, cage, and hygiene management, contribute to the imbalance of the normal flora, hence allowing pathogens to dominate the microbiome [1]. According to Lowe *et al*. [2], nasal cavity and tonsil of a healthy pig contain equal proportions of Gram-positive and Gram-negative bacteria. Furthermore, Baele *et al*. [3] discovered over 30 different species of Gram-positive bacteria in the nasal cavity and tonsil of a pig. Several Gram-positive bacteria, such as *Streptococcus* spp., *Enterococcus* spp. *Lactobacillus* spp., and *Staphylococcus* spp., can threaten pig productivity [1,3,4]. *Streptococcus equi* subspecies *zooepidemicus* was identified as the causative agent of the meningitis outbreak, which resulted in the deaths of thousands of pigs in Bali in 1994 [5]. Based on biochemical and serological tests, this organism was classified as part of the *Streptococcus* b-hemolytic Group C. Furthermore, this disease resulted in economic losses due to its attack on monkeys in several tourism forests in Bali [6]. Recently, a human meningitis outbreak was also reported after the consumption of pork-derived foods at Sibang Kaja, Badung-Bali [7]. However, the strain of the causative agent obtained from the pigs’ nasal has not been completely identified.

In general, the conventional identification of microorganisms involves culturing the agent in a specific medium and then analyzing its physiological and biochemical characteristics. The culture method is the gold standard for conventional identification [8,9]. However, several limitations include its inapplicability to fastidious microorganisms and inability to classify species levels that are phenotypically confusing or have not been discovered using biochemical test results [8].

Recently, molecular-based identification methods with rapid speed and a high level of sensitivity as well as specificity have been developed [10]. Furthermore, several scientists have employed molecular techniques, such as using the 16S ribosomal RNA (rRNA) gene as a target to classify and characterize bacteria. This method has also been successfully used in the analysis of *Pasteurella multocida* [11] and as an accurate and specific technique in the identification of *S. equi* subspp. *zooepidemicus* [12], in the analysis of *Escherichia coli* O157:H7 [13]. This study aimed to identify and characterize Gram-positive β-hemolytic organisms collected from nasal swab of pigs from the outbreak area, as well as to compare API Kit and *16S rRNA* gene analysis methods.

## Materials and Methods

### Ethical approval

An approval from the Institutional Animal Ethics Committee was not required to conduct this study, as no live animals were used. Furthermore, only two isolates, which were stored in a freezer at –20°C, were used.

### Study period and location

This study was conducted from April to November 2019 in the Laboratory of Veterinary Public Health, Faculty of Veterinary Medicine, Udayana University.

### Bacterial isolates

The two bacterial isolates used in this study were Punggul Swab Nasal No.2 (PSN 2) and PSN 19, which were obtained from the nasal cavity of pigs. Furthermore, according to a previous study, both isolates were Gram-positive b-hemolytic organisms and originated from the human meningitis outbreak in Badung Regency, Bali-Indonesia [7].

### Cultivation of isolates

Isolates were first cultured in a 5% defibrinated sheep blood agar plate and then incubated at 37°C for 24 h. Subsequently, Gram staining, catalase, oxidase, salt tolerance (6.5% NaCl), and hemolysis tests were conducted on some suspected colonies [14,15].

### Identification of isolates using Kit API 20 Strep test

The isolates were biochemically identified using Kit API 20 Strep (Biomerieux, France) according to the manufacturer’s instructions with slight modification [16]. A bacterial colony grown on the defibrinated sheep blood agar plate was then transferred briefly into a tube containing 2 mL of distilled water. Using a Pasteur pipette (Sugitech, Indonesia), three drops of suspension were then placed into each microcapsule of the strips supplied in the kit. Afterward, these strips were incubated at 37°C for 4 h. The reagents were then added and the strips were exposed to strong light for reading enzymatic activities. The test results were then recorded, and the strips were incubated at 37°C for 20 h. The manufacturer’s profile index and table were used to interpret the test results for the bacterial species identification obtained at 4 and 24 h of incubation.

### Deoxyribonucleic acid (DNA) extraction and polymerase chain reaction (PCR) amplification of 16S *rRNA* gene

According to the manufacturer’s procedure with slight modification, the Geneaid Kits (Presto Mini gDNA Bacteria Cat. GBB100) were used to extract the DNA of all the isolates. Furthermore, the universal primer B27F (5’-AGAGTTTGATCCTGGCTCAG-3’) and U1492R (5’-GGTTACCTTGTTACGACTT-3’) were used to analyze the *16S rRNA* [13]. The PCR program was conducted with 36 μL reaction volume containing 2 μL DNA template, 25 μL My Taq HS Red Mix, 7 μL distilled water, and 1 μL (20 pmol/mL) of primer 27F and U1492R each. The PCR amplification was performed by an initial DNA denaturation at 94°C for 5 min, and then by 30 cycles of denaturation at 94°C for 1 min. This procedure continued with annealing at 45°C for 45 s and an extension at 72°C for 1 min. The amplification process was completed by a final extension at 72°C for 5 min. Furthermore, 5 μL of PCR product was analyzed by electrophoresis in 1% agarose [13].

### Sequencing and phylogenetic analysis

The process of sequencing the isolates’ *16S rRNA* gene was conducted with a genetic analyzer (ABI Prism 3130 and 3130xl Genetic Analyzer, Thermo Fisher Scientific, USA) at Singapore through the Institute for sequencing service providers at PT Genetika Science, Jakarta. Furthermore, similar primers with the previous PCR reactions were used. The sequences were edited to exclude the PCR primer binding sites and corrected with the MEGA 5.2 version software (https://www.megasoftware.net/) [13]. Furthermore, the full gene sequences were compared automatically using the BLAST program against the sequences of bacteria available in databanks (www.ncbi.nlm.nih.gov). The neighbor-joining algorithm method was then used to construct the phylogenetic tree [16-18].

## Results

The cultivation of isolates resulted in the growth of bacteria in the blood agar medium. This development was characterized by the presence of white colonies with 1.5×2 mm diameter, and coccus Gram-positive organisms in the form of paired or short chains. The catalase and oxidase test results were negative while the salt tolerance test (6.5% NaCl) was positive, with β hemolysis in the blood agar medium. Moreover, β hemolysis bacteria are pathogenic and capable of breaking down red blood cells.

Table-1 illustrates that the cultivation of both isolates was in line with the biochemical identification results using the Kit API 20 Strep test. Table-1 shows the biochemical identification results, where the PSN 2 and 19 isolates were identified as *Enterococcus faecium*. The Kit API 20 Strep is a biochemical identification kit involving 20 sugar tests. This analysis can identify a broad group of *Streptococcus* and *Enterococcus* bacteria using dehydrated substrates to detect enzymatic activity and fermentation of sugars [19,20]. Based on Table-1, the Kit API 20 Strep test results illustrated that only PSN 2 isolates fermented b-galactosidase (b-GAL). However, the presence of inulin acid fermentation (INU) was observed in both isolates.

**Table-1:** The results biochemical identification by the Kit API 20 strep of PSN 2 and PSN 9.

Test	Reaction	Bacterial isolates	

		PSN 2	PSN 19
VP	Acetoin production	+	+
HIP	Hippurate	–	–
ESC	Aesculin hydrolysis	+	+
PYRA	Pyrrolidonylaryl-amidase	+	+
αGAL	α-galactosidase	–	–
βGUR	β-glucuronidase	–	–
βGAL	β-galactosidase	+	–
PAL	Alkaline phosphatase	+	+
LAP	Leucine arylamidase	+	+
ADH	Arginine dihydrolase	+	+
RIB	Ribose fermentation	+	+
ARA	Arabinose fermentation	+	+
MAN	Mannitol fermentation	+	+
SOR	Sorbitol fermentation	+	+
LAC	Lactose fermentation	+	+
TRE	Trehalose fermentation	+	+
INU	Inulin fermentation	+	+
RAF	Raffinose fermentation	+	+
AMD	Starch fermentation	+	+
GLYG	Glycogen fermentation	+	+
βHAEM	Hemolysis	+	+
Bacterial species		*E. faecium*	*E. faecium*

*E. faecium=Enterococcus faecium*, PSN=Punggul Swab Nasal

**Figure-1:**
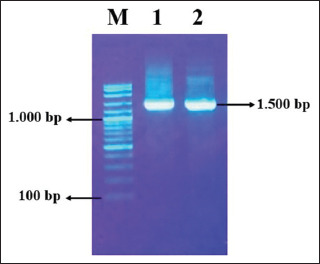
Amplification of the 16S ribosomal RNA gene of Gram-positive β hemolytic bacteria isolated from nasal of pig in 1% agarose. M: Marker 1kb, 1: PSN 2 isolate, 2: PSN 19 isolate. PSN=Punggul Swab Nasal.

Figure-1 depicts the successful amplification of the *16S rRNA* gene target to confirm the Kit API 20 Strep result. This finding was characterized by the appearance of a single band in the 1500 bp position, which corresponded with the primers’ gene target.

According to Table-2, the success of the *16S rRNA* gene amplification was preceded by sequencing. Subsequently, the results of the nucleotides sequence were used to calculate the genetic distance. The pairwise distance data in Table-2 show that both local isolates (PSN 2 and PSN 19) had a high degree of similarity (98.3%). These isolates were also closely related to *E. faecium* (NR042054), with 99 and 98.1% for PSN 2 and PSN 19, respectively, or a 10 and 19 nucleotides difference from 1000 nucleotides pairwise.

**Table-2:** The pairwise distance among the Gram-positive β hemolytic bacteria isolated from nasal of pig compared to several nucleotide sequences accessed in GenBank.

	PSN _2	PSN_19	*En terococcus faecium*_NR042054	*Bacillus thuringiensis*_MN420977	*Bacillus cereus*_KF475850	*Streptococcus zooepidemicus*_NR036758	*Stre ptococcus pyogenes*_NR028598	*Stre ptococcus porcinus*_NR024634	*Stre ptococcus agalactiae*_NR040821	*Sta phylococcus hemolyticus*_JF799877	*Sta phylococcus aureus*_NR118997	*Aerococcus viridans*_NR118723
PSN_2												
PSNs_19	0.017											
*Enterococcus faecium*_NR042054	0.010	0.019										
*Bacillus thuringiensis_*MN420977	0.072	0.075	0.075									
*Bacillus cereus*_KF475850	0.072	0.075	0.075	0.000								
*Streptococcus zooepidemicus*_NR036758	0.126	0.133	0.123	0.156	0.156							
*Streptococcus pyogene*_NR028598	0.106	0.111	0.106	0.142	0.142	0.050						
*Streptococcus porcinus*_NR024634	0.107	0.113	0.104	0.148	0.148	0.050	0.052					
*Streptococcus agalactiae_*NR040821	0.099	0.104	0.099	0.138	0.138	0.058	0.031	0.028				
*Staphylococcus hemolyticus_*JF799877	0.073	0.080	0.072	0.061	0.061	0.163	0.139	0.143	0.130			
*Staphylococcus aureus*_NR118997	0.073	0.080	0.072	0.057	0.057	0.163	0.139	0.143	0.130	0.003		
*Aerococcus viridans*_NR118723	0.070	0.069	0.068	0.088	0.088	0.161	0.147	0.136	0.131	0.086	0.086	

PSN=Punggul Swab Nasal

**Figure-2:**
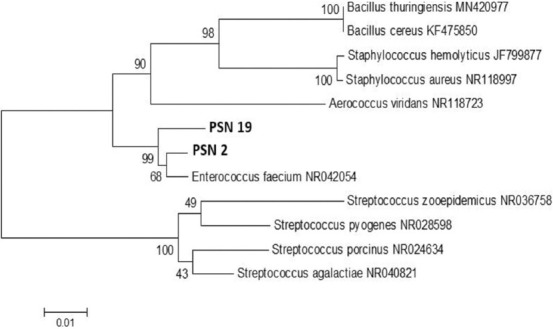
The phylogenetic tree of PSN 2 and PSN 19 Gram-positive β hemolytic isolated from nasal of pig based on 16S ribosomal RNA gene sequences. PSN=Punggul Swab Nasal.

Figure-2 illustrates that the results of the data in Table-2 can be used to construct their phylogenetic tree. On further analysis, the phylogenetic tree in Figure-2 grouped both isolates into one *E. faecium* (NR042054) clade, based on the data in Table-2.

## Discussion

According to the cultivation results, both bacterial isolates belonged to the *Enterococcus* spp. Baele *et al*. [3] discovered above 30 different species of Gram-positive bacteria in the nasal cavities and tonsils of pigs, including those in the *Streptococcus*, Staphylococcus, *Enterococcus*, and Lactobacillus genera. Furthermore, Manero and Blanch [21] stated that *Enterococcus* genus is a group of Gram-positive bacteria D-*Streptococcus*, which exists in the form of a single coccus, paired or short-chain, negative oxidase, negative catalase, non-spore producing, and facultative anaerobes. This organism is also able to ferment carbohydrates into lactic acid, grow optimally in an environment with 30-37°C, 6.5% NaCl, and pH 9.6, as well as exhibit either a, b or g blood hemolysis.

Moreover, the Kit API 20 Strep with 20 sugars test highlighted that the PSN 2 and PSN 19 isolates were *E. faecium*. The results were consistent with the data in the API 20 STREP Profile Index/Identification table and with the study by Pelinescu *et al*. [22]. This finding showed that *E. faecium*, which was identified by the API 20 Strep produced b-GAL, but not inulin acid (INU).

To confirm the Kit API 20 Strep test results, the identification process was followed by molecular analysis, according to the method stated previously [13]. The *16S rRNA* gene analysis result also supported the biochemical test, which identified both isolates as an *Enterococcus* spp. Angeletti *et al*. [23] identified 253 isolates from the 279 clinically isolated specimens using commercial kits (API 32 Strep and API 20 Strep) and molecular methods. Although no commercial kit included the whole test set, some were in the enzyme activity-based kits that might be used with the proposed key. This key was designed for use in routine applications, specifically in environmental and clinical studies with many isolates [24].

Subsequently, molecular-based identification methods have a high level of sensitivity and specificity [25]. The development of molecular biological techniques has allowed rapid and reliable diagnosis of infections caused by bacteria of the *Streptococcus* and *Enterococcus* genera [26].

The *16S rRNA* gene sequencing has proven to be an effective tool in the classification of microorganisms, including streptococci and enterococci [25,27]. This process addresses the need for a more precise and accurate method of diagnosis in microbiology to supplement conventional biochemical, microbiological approaches, which have several advantages. The rRNA coding gene is the most conserved gene with a sustainable structure, allowing the *16S rRNA* to be used in PCR and sequencing analysis [25]. Furthermore, the queries were categorized as the same species if the *16S rRNA* gene sequences were more than 90% comparable, the nucleotides differ between 14 and 22 bp queries, or the nucleotide percentage difference is between 1 and 1.5% [13,28]. Janda and Abbott [9] also recommended a concept of similarity, which includes (i) the *16S rRNA* gene length should be at least between 500 to 525 bp and ideally 1300 to 1500 bp and (ii) the criteria for species identification should have a minimum of >99% similarity and ideally >99.5%.

Based on the above concept, both PSN 2 and PSN 19 isolates were confirmed as *E. faecium* (NR042054), with 68% bootstrap. Furthermore, this study’s results are supported by prior findings [28], which stated a significant level of similarity in the nucleotide sequence of the *16S rRNA* gene sequences. Therefore, Enterococci, which are derived from the same species, tend to provide a high bootstrap value in the analysis of kinship [20]. According to Bertelloni *et al*. [29] and Castillo-Rojas *et al*. [30], *E. faecium* and *Enterococcus faecalis* are the most prevalent organisms observed in the gastrointestinal flora of warm-blooded animals, including pets, wild animals, and humans. The bacterium can contaminate soil and water [31] and has also been isolated from the feces of reptiles, birds, and insects. Moreover, bacteria are easily isolated from the environment inhabited by the host due to their extensive appearance in humans and animals [32,33]. These organisms are characterized by their ability to resist harsh conditions and the presence of a “Janus face” behavior, which allows them to transform from a commensal into a causative agent of invasive infections [34].

*E. faecalis* and *E. faecium* are the two most pathogenic enterococcal species to humans, with the highest resistance to desiccation and starvation. Moreover, vancomycin-resistant Enterococci are globally distributed, with approximately 80% of *E. faecium* isolates being resistant in some hospitals [35]. These organisms are majorly isolated from the nasal cavity and tonsils of piglets. In addition, enterococci species are termed opportunistic bacteria and can cause severe infections and obtain, express, and transfer antimicrobial resistance. Meningitis, bacteremia, and endocarditis in pigs, all are caused by the *Enterococcus* pathogen [3].

For several years, *E. faecium* was considered a digestive tract commensal, which only sporadically triggers opportunistic infections in severely ill patients. However, vancomycin-resistant *E. faecium* has emerged globally over the last two decades, as an important cause of nosocomial infections, specifically in immunocompromised patients. Molecular epidemiological studies of both human and animal-derived *E. faecium* isolates revealed the existence of host-specific genogroups, including a genetic lineage designated CC17, associated with nosocomial infections [36]. Furthermore, E. faecium bacteremia may result in sepsis in immunocompromised patients, with a high mortality rate. Therefore, careful pathogen detection and early initiation of treatment are essential for positive patient outcomes [37]. Streptococci and enterococci are also significant opportunistic pathogens in epidemiology and infectious medicine. In addition, the most challenging aspects of species identification include the high genetic and taxonomic similarities and several reclassifications within a genus [38].

## Conclusion

The local isolates PSN 2 and PSN 19 as Gram-positive bacteria with β-hemolysis activity originated from the nasal swab of pigs were identified as *E. faecium*. This result was based on the biochemically and molecularly test, both isolates are pathogenic to humans, so they are interesting for further study, especially in the zoonotic aspect.

## Authors’ Contributions

KJPP and IWS: Conceived, designed, and supervised the study. IDMS and IBNS: Analyzed the data and edited the final manuscript. RKP: Collected the isolates and performed laboratory activities. All authors read and approved the final manuscript.
